# Ranking attention multiple instance learning for lymph node metastasis prediction on multicenter cervical cancer MRI

**DOI:** 10.1002/acm2.14547

**Published:** 2024-10-06

**Authors:** Shan Jin, Hongming Xu, Yue Dong, Xiaofeng Wang, Xinyu Hao, Fengying Qin, Ranran Wang, Fengyu Cong

**Affiliations:** ^1^ Cancer Hospital of Dalian University of Technology Dalian University of Technology Shenyang China; ^2^ School of Biomedical Engineering, Faculty of Medicine Dalian University of Technology Dalian China; ^3^ Liaoning Key Laboratory of Integrated Circuit and Biomedical Electronic System Dalian University of Technology Dalian China; ^4^ Dalian Key Laboratory of Digital Medicine for Critical Diseases Dalian University of Technology Dalian China; ^5^ Department of Radiology, Cancer Hospital of China Medical University Liaoning Cancer Hospital and Institute Shenyang China; ^6^ Department of Quantitative Health Sciences Cleveland Clinic Cleveland Ohio USA; ^7^ Faculty of Information Technology University of Jyvaskyla Jyvaskyla Finland; ^8^ Key Laboratory of Social Computing and Cognitive Intelligence, Dalian University of Technology Ministry of Education Dalian China

**Keywords:** cervical cancer, deep learning, lymph node metastasis, magnetic resonance imaging, multiple instance learning

## Abstract

**Purpose:**

In the current clinical diagnostic process, the gold standard for lymph node metastasis (LNM) diagnosis is histopathological examination following surgical lymphadenectomy. Developing a non‐invasive and preoperative method for predicting LNM is necessary and holds significant clinical importance.

**Methods:**

We develop a ranking attention multiple instance learning (RA‐MIL) model that integrates convolutional neural networks (CNNs) and ranking attention pooling to diagnose LNM from T2 MRI. Our RA‐MIL model applies the CNNs to derive imaging features from 2D MRI slices and employs ranking attention pooling to create patient‐level feature representation for diagnostic classification. Based on the MIL and attention theory, informative regions of top‐ranking MRI slices from LNM‐positive patients are visualized to enhance the interpretability of automatic LNM prediction. This retrospective study collected 300 female patients with cervical cancer who underwent T2‐weighted magnetic resonance imaging (MRI) scanning and histopathological diagnosis from one hospital (289 patients) and one open‐source dataset (11 patients).

**Results:**

Our RA‐MIL model delivers promising LNM prediction performance, achieving the area under the receiver operating characteristic curve (AUC) of 0.809 on the internal test set and 0.833 on the public dataset. Experiments show significant improvements in LNM status prediction using the proposed RA‐MIL model compared with other state‐of‐the‐art (SOTA) comparative deep learning models.

**Conclusions:**

The developed RA‐MIL model has the potential to serve as a non‐invasive auxiliary tool for preoperative LNM prediction, offering visual interpretability regarding informative MRI slices and regions in LNM‐positive patients.

## 1 INTRODUCTION

Cervical cancer is one of the most prevalent cancers affecting the female reproductive system worldwide.^1^ The main treatment for cervical cancer includes surgery, chemotherapy, and radiotherapy.^2^ Preoperative examination is a reliable basis for surgery in cervical cancer according to the International Federation of Gynecology and Obstetrics (FIGO) guideline.^3^ Lymph node metastasis (LNM) is a critical prognostic factor for the overall survival and recurrence of cervical cancer. In the current clinical diagnostic process, histopathological examination following surgical lymphadenectomy provides the basis for LNM diagnosis. However, surgical procedures are invasive and time‐consuming, with potential postoperative risks. An accurate, noninvasive, and preoperative method for early LNM diagnosis is necessary and meaningful for selecting suitable treatment protocols and surgical procedures. Magnetic resonance imaging (MRI) is an ideal imaging protocol due to its high imaging resolution and non‐invasiveness. MRI can provide abundant information about cervical cancer, such as its location, shape, boundary, and texture. Some studies have demonstrated that MRI possesses the potential to be utilized for the preoperative diagnosis of LNM.^4^, ^5^, ^6^ Thus, it would be promising to develop an advanced LNM diagnostic model based on cervical cancer MRI to provide precise diagnosis and treatment decisions.

Some previous studies explored feature‐based approaches to perform automatic diagnosis of cervical cancer based on MRI data.^7^, ^8^, ^9^ Feature‐based approaches typically consist of two modules: radiomics feature extraction and diagnostic classification. These methods heavily rely on manual annotations of regions of interest (ROI) for radiomics feature extraction. Manual annotations are time‐consuming and heavily dependent on expert domain knowledge, which impedes their utilizations on new unannotated data. Other cervical cancer studies aim to construct deep learning classification models.^10^, ^11^, ^12^ These studies perform diagnostic prediction at MRI slice‐level, treating every 2D MRI image as a separate sample. However, since not all slices of a positive patient necessarily include pertinent information, this could result in misleading false positive results.^13^

Recently, convolutional neural networks (CNNs) that can automatically detect informative imaging features have been widely explored in automatic disease diagnosis.^14^, ^15^, ^16^ However, it is challenging to directly apply CNN on 3D MRI analysis. Feeding 3D MRI to a deep learning model requires a relatively large graphics processing unit (GPU) memory usage and high‐performance hardware resources.^17^ Besides, training a 3D CNN model is a difficult task due to the excessive number of trainable parameters and high complexity.^18^ To effectively learn features from weakly annotated 3D MRI data for making LNM status predictions, multiple instance learning (MIL) is a promising solution as it can use 2D CNN with fewer parameters and lower complexity to learn imaging features in instance‐level and then make predictions in bag‐level. Multiple instances convolutional neural network (MICNN) is a combination of MIL and CNN and has been applied to various diagnostic tasks, including Alzheimer's disease diagnosis,^19^ COVID‐19 severity assessment,^16^ abnormal cell detection,^20^ and microsatellite instability prediction.^21^ The MIL pooling function is a key component in MICNN, playing a crucial role in fusing instance‐level features into a bag‐level representation. To accommodate more complex application scenarios, MIL pooling has evolved from rule‐based pooling, such as mean pooling, max pooling, and contextual MIL pooling layer (C‐MPL),^14^ to attention‐based pooling, such as attention MIL,^22^ gated‐attention MIL,^22^ and Double‐Tier Feature Distillation MIL (DTFD‐MIL).^23^

Inspired by the above existing studies, we propose a ranking attention multiple instance learning (RA‐MIL) model to diagnose LNM using cervical cancer MRI. The RA‐MIL model performs patient‐level prediction with a reduced computational burden. Meanwhile, with the help of CNN, the RA‐MIL model automatically captures task‐related features without using manual tumor annotations. Our main contributions are summarized as follows: (1) We propose an end‐to‐end RA‐MIL model for LNM diagnosis using both internal and public cervical cancer MRI, demonstrating promising performance even in the absence of prior manual tumor annotations. (2) A ranking attention pooling is designed to aggregate informative imaging features, which helps improve prediction performance and provide the interpretability of automatic LNM diagnosis. (3) We perform experiments across internal and public datasets from multiple centers, demonstrating improvements in LNM prediction over comparative methods.

## 2 MATERIALS AND METHODS

### 2.1 Datasets and preprocessing

This retrospective study was reviewed and approved by the Institutional Review Board, and the requirement for informed consent was waived. We collected 306 cervical cancer patients with T2‐weighted imaging (T2) MRI from Liaoning Cancer Hospital and Institute. Patients were included based on the following criteria: (1) histologically confirmed cervical cancer; (2) preoperative pelvic MRI, including plain scan and enhanced MRI; (3) no antineoplastic therapy before the pelvic MRI examination; (4) patients who underwent radical hysterectomy with pelvic lymph node dissection and received postoperative adjuvant treatment. The exclusion criteria were as follows: (1) other histological types except adenocarcinoma, squamous cell carcinoma, and adeno‐squamous carcinoma; (2) MRI images deemed non‐evaluable due to artifacts or other factors; (3) patients with other concurrent malignant tumors. MRI scans were performed by using a 3.0T unit MRI scanner (Magnetom Trio, Siemens Medical Solutions, Germany) with an 8‐channel phased array body coil and respiratory gating technology. Patients were in the supine position during the MRI acquisition. Axial fat suppression fast spin‐echo T2‐weighted (T2W) images were used in this study. LNM in all enrolled cases occurred in the lateral pelvic wall, particularly in the internal iliac and obturator lymph nodes.

**FIGURE 1:**
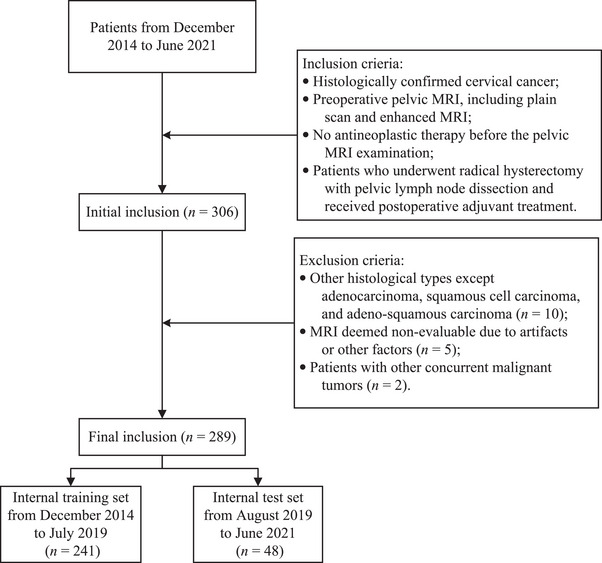
The workflow diagram of patient recruitment.

According to different scanning times, our internal dataset is divided into two sets: a training set with 241 patients collected from December 2014 to July 2019, and a test set with 48 patients collected from August 2019 to June 2021. The workflow of patient recruitment is shown in Figure 1. All ground truth LNM labels are acquired from clinical diagnoses made by pathologists.

Additionally, we attempted to collect a public test dataset from The Cancer Genome Atlas (TCGA) cervical squamous cell carcinoma and endocervical adenocarcinoma (CESC) project available on The Cancer Imaging Archive (TCIA) (https://www.cancerimagingarchive.net). This dataset comprises a total of 54 cases. However, as 43 cases in the TCIA lack LNM labels, only 11 patients from this source are used in the public test dataset.

The distribution of clinicopathological characteristics of patients included in our research is displayed in Table 1. The ages of patients are presented with the mean age (the minimum age, the maximum age). Portions of positive LNM patients in the training set, test set, and public set are 39.4%, 41.7%, and 27.2%, respectively.

**TABLE 1:** The distribution of clinicopathological characteristics across our datasets.

Variable	Training cohort (*n* = 241)	Internal test cohort (*n* = 48)	Public test cohort (*n* = 11)
A. Clinical characteristics			
Age (year)	51.6 (25.0, 75.0)	53.8 (30.0, 67.0)	45.1 (26.0, 72.0)
B. Pathological characteristics			
LNM status			
Positive	95 (39.4%)	20 (41.7%)	3 (27.2%)
Negative	146 (60.6%)	28 (58.3%)	8 (72.8%)

Abbreviation: LNM, lymph node metastasis.

As part of the preprocessing, we convert each 3D T2W volume in DICOM format into a sequence of 2D MRI slices saved in the PNG format. To accommodate the input size of the deep learning model and exclude irrelevant regions (i.e., image boundary regions without pelvic tissue) for LNM prediction in MRI images, a centering patch with the size of 224 × 224 pixels is cropped from each MRI slice. Furthermore, tumor regions of MRI slices in our internal dataset are manually annotated by two experienced radiologists, although our RA‐MIL model does not rely on any tumor annotations. These tumor annotations are only used to implement existing methods for comparison.

### 2.2 Ranking attention multiple instance learning (RA‐MIL) model

Since our LNM diagnostic labels are directly derived from clinical reports of cervical cancer patients, there are no MRI slice‐level labels or annotations. This poses a great challenge for developing fully‐supervised deep learning models. In this study, we introduce a MIL framework to perform patient‐level LNM prediction based on weakly‐annotated MRI data. Specifically, the 3D MRI data with *K* slices is considered as a bag, that is, *X* = {*x*_1_, *x*_2_,…, *x_K_*}, where *K* could vary in different bags because of various scan slice thicknesses, and *x_k_*, 1≤k≤K, represents the *k*th MRI slice (or instance) in the bag. Each bag has a single binary label *Y*, where *Y* ∈ {0,1}, which depends on the histological diagnosis provided by pathologists. Instances within a bag have individual binary labels, that is, {*y*_1_,*y_2_* …, *y_K_*}, but their labels are unknown during training.

**FIGURE 2:**
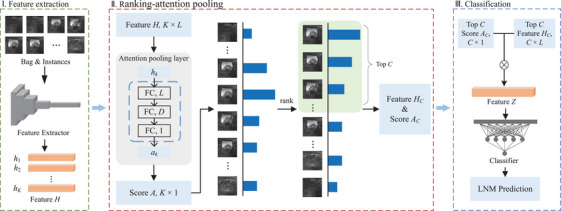
Illustration of the proposed ranking attention multiple instance learning (RA‐MIL) model. The RA‐MIL model consists of three stages: feature extraction, ranking‐attention pooling, and classification. LNM: lymph node metastasis.

Figure 2 shows the overview of our designed RA‐MIL model for LNM diagnosis. It is observed in Figure 2 that the RA‐MIL model contains three stages: feature extraction, ranking attention pooling, and classification, as highlighted with the green, red, and blue dashed rectangles, respectively.

In Stage I, we first perform slice‐level feature extraction from the 3D MRI, where the CNN serves as the feature encoder to automatically extract low‐level and fine‐grained features from MRI slices. In this study, the pre‐trained ResNet50 architecture, excluding classification layers, is applied to extract MRI slice‐level features from the cropped patches of 2D MRI slices. Assume that the feature extraction network is symbolized as *f_e_* (•), where the last layer is a full connection layer with *L* neuron nodes. The feature extraction for the 3D MRI *X* is represented as:

(1) $$\begin{array}{c} H=f_{e}\left(X\right) \end{array}$$

where *H* ∈ ℝ*^K^* ^×^ *^L^* represents the encoded feature matrix for *K* MRI slices. Each MRI slice is encoded into a *L* dimensional feature vector (e.g., *L = *1000).

In Stage II, the ranking attention pooling is developed to build the connection between bags and their instances in MIL. Firstly, because of the trainable and self‐adaptive characteristics, attention pooling is adopted to compute instance‐level weighting scores. With *H* = {*h*_1_,…,*h_K_*} as the input to the attention pooling layer, weighting scores *A* = {*a*_1_,…,*a_K_*} are learned by training a neural network with a layer of *D* neuron nodes following an *tanh* activation function and a layer of 1 neuron node (see Figure 2). *a_k_* is formally calculated as:

(2) $$\begin{array}{c} a_{k}=\frac{exp\left\{w^{T}tanh\left(Vh_{k}^{T}\right)\right\}}{\sum_{j=1}^{K}exp\left\{w^{T}tanh\left(Vh_{j}^{T}\right)\right\}} \end{array}$$

where *w* ∈ ℝ*^D^* ^×1^ and *V* ∈ ℝ*^D^* ^×^ *^L^* are trainable parameters. The weights are normalized by the *softmax* function to ensure that the sum of weights equals to 1. Additionally, *a_k_* can be considered as the positive probability of instances. Given that instances from a bag shows different diagnostic information, we design a top‐ranking layer to select more informative MRI slices according to the sequence of *a_k_*. By sorting the sequence *a_k_* in descending order, we select the top *C* attention scores *A_C_* = {*a*_1_,…,*a_C_*} and their corresponding instance‐level feature representations *H_C_* = {*h*_1_,…,*h_C_*}. The ranking attention pooling is then computed as:

(3) $$\begin{array}{c} z=\sum_{c=1}^{C}a_{c}h_{c} \end{array}$$

where *z* ∈ ℝ^1×^ *^L^* is the bag‐level feature representation. Note that *C* is experimentally set as 10 in our evaluations. The selected attention scores *A_C_* are not rescaled during bag‐level feature aggregations, ensuring that the original distribution of these scores, as learned by the attention pooling mechanism, is maintained.

In Stage III, the feature vector *z* is fed into a classification head consisting of a layer of one neuron node and the sigmoid activation function, which outputs the bag‐level (i.e., patient‐level) LNM status prediction. To tackle the class imbalance problem in LNM prediction, a weighted binary cross entropy loss *L* is adopted to optimize parameters in our RA‐MIL model, that is,

(4) $$\begin{array}{ccc} L & = & \beta_{P}\sum_{Y=1}-p\left(X\right)\log q\left(X\right)\\ & & +\beta_{N}\sum_{Y=0}-\left(1-p\left(X\right)\right)\log\left(1-q\left(X\right)\right) \end{array}$$

(5) $$\begin{array}{c} \beta_{P}=\frac{N}{P+N},\beta_{N}=\frac{P}{P+N} \end{array}$$

where *p*(*X*) and *q*(*X*) are ground truth and prediction labels, respectively; *P* and *N* represent the number of positive and negative labels, respectively; *β*_P_ and *β_N_* are the weights of positive and negative categories, respectively.

### 2.3 Implementation and evaluation metrics

To get a preliminary evaluation, a 5‐fold cross‐validation is conducted on our internal training set. The training set is randomly partitioned into five subsets with the same proportion of positive versus negative LNM status as the whole training dataset. It is found that our RA‐MIL model demonstrates promising LNM prediction performance from T2 MRI, achieving an AUC value of approximately 0.75 in internal cross‐validation (see Figure S1). After that, the internal training set is fully utilized to train the RA‐MIL model. The internal test and public sets are then utilized to thoroughly assess the generalization capability of our model.

Our RA‐MIL model is implemented in Python with PyTorch (version 1.7.1) on a server equipped with two NVIDIA RTX3090 GPUs. The model is trained in an end‐to‐end manner, using a pre‐trained ResNet50 as the initial feature extractor, which is then fine‐tuned along with the pooling operation and classification head. The training is conducted by utilizing the Adam optimizer with a learning rate of 2 × 10^−5^, *β*_1_ = 0.9, *β*_2_ = 0.999, and weight decay of 1 × 10^−4^. We set the batch size as 1. Note that 10% of the subjects of the training set are randomly used as a validation set to monitor the training process. The training continues for a maximum of 200 epochs, with early stopping implemented if the *AUC* value on the validation set does not increase for 20 consecutive epochs. To prevent over‐fitting and obtain a more generalizable model, data augmentations are utilized on the training set, which includes random horizontal and vertical flipping with a probability of 0.5, as well as random rotation with a probability of 0.2 and a range of 0° to 40°. Besides the *AUC* value, the accuracy (*Acc*), precision (*Pre*), recall (*Rec*), specificity (*Spe*), and F1‐score (*F1*) are also utilized as evaluation metrics.^24^ Mann–Whitney *U* test is applied as the statistical test to compare RA‐MIL with other SOTA MIL methods. Our source codes and demo data of the TCGA CESC project are publicly available at https://github.com/miscut/RA‐MIL.

## 3 RESULTS

### 3.1 Evaluations for model selection

To assess the impact of various configurations in our RA‐MIL model, we first conduct evaluations for model selection using our internal test set.

#### 3.1.1 Effect of different CNN backbones

**FIGURE 3:**
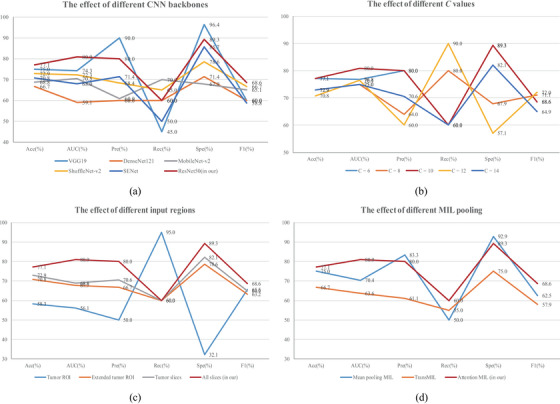
Results of model selection on internal test set. The effect of (a) different CNN backbones; (b) different *C* values; (c) different MRI input regions; and (d) different MIL pooling operations. Acc, accuracy; AUC, area under the receiver operating characteristic curve; Pre, precision; Rec, recall; Spe, specificity; F1, F1‐score.

Different CNN backbones can be used as feature extractors in our RA‐MIL model. In this study, we compare the ResNet50 backbone with five other SOTA CNN backbones, including VGGNet19,^25^ DenseNet121,^26^ MobileNet‐v2,^27^ ShuffleNet‐v2,^28^ SENet.^29^ These CNN models have been used in many medical image classification tasks.^24^, ^30^ For a fair comparison, we maintain consistent data preprocessing and partition strategies across all experiments. Figure 3a lists the results of using different CNN backbones. It is observed in Figure 3a that the ResNet50 and SENet provide overall better performances than other CNN backbones. In particular, ResNet50 achieves the best results on three evaluation metrics, with *Acc* = 0.771, *AUC* = 0.809, and F1 = 0.686. Given the imbalance in the distribution of LNM positive and negative cases (see Table 1), it is crucial to consider not only *Acc* but also *AUC* and *F1* scores as key criteria for selecting the feature extractor. These metrics provide insight into the confidence and true prediction rates for different classes in an imbalanced classification task. Thus, the ResNet50 backbone is utilized for feature extraction in our LNM prediction task.

#### 3.1.2 Effect of different top‐ranking values

We hypothesize that MRI slices play different roles in LNM prediction. The middle slices in the MRI sequence tend to contain more information about tumor regions, whereas the marginal slices with fewer tissues are usually uninformative to LNM diagnosis, which may bring in noise and increase the risk of overfitting. As such, a ranking attention strategy is designed to exclude imaging features of uninformative MRI slices. Specifically, the top‐*C* feature vectors of MRI slices are aggregated to create the patient‐level feature representation (see Equation (3)). Considering that the 3D MRI in our dataset has 20 slices per patient, a series of *C* values ranging from 6 to 14 with a step of 2 are tested to evaluate the impact of top‐ranking values. Figure 3b shows the evaluation results. As observed in Figure 3b, the overall better performance is achieved when *C *= 10, where the highest *AUC* value of 0.809 is reached. This suggests that adaptive utilization of half of the MRI slices could improve the performance in our LNM prediction.

#### 3.1.3 Effect of different MRI input regions

Our collaborating radiologists have manually delineated tumor regions on the MRI slices. To evaluate the impact of different MRI input regions, we perform an ablation study by utilizing the patches of tumor ROI,^12^ extended tumor ROI, only tumor slices, and all MRI slices as our model inputs, respectively. Note that the patches of tumor ROI are cropped by following the bounding rectangles of manually annotated tumor regions. The extended tumor ROI incorporates 30 more pixels around tumor ROI by following the reference,^31^ and then the patches of extended tumor ROI are cropped by following the bounding rectangles as well. The patches of only tumor slices or all MRI slices are cropped from central regions of MRI slices and then used as inputs for our model. Figure 3c shows the results with different MRI input regions to our RA‐MIL model. It is observed in Figure 3c that using all MRI slices without relying on any prior tumor annotations achieves the overall best performance, with almost the highest values for all evaluation metrics. These results suggest that informative regions for predicting cervical cancer LNM status may extend beyond tumor regions alone. In other words, peritumoral tissues and other normal tissues in T2 MRI slices could also contribute to distinguishing LNM positive and negative status for cervical cancer patients.

#### 3.1.4 Effect of different MIL pooling

To further evaluate different MIL pooling methods on a set of ranked slices, selected instance‐level features by ranking rules are separately fed into attention MIL, mean pooling MIL, and TransMIL^32^ to generate the bag‐level features for LNM prediction. Figure 3d shows the results of different MIL pooling methods. The RA‐MIL model with attention MIL performs the best, as it provides trainable attention weights that enhance model performance. Besides, compared to the TransMIL, our RA‐MIL has fewer parameters, which helps mitigate the risk of overfitting.

### 3.2 Comparison with other methods

**FIGURE 4:**
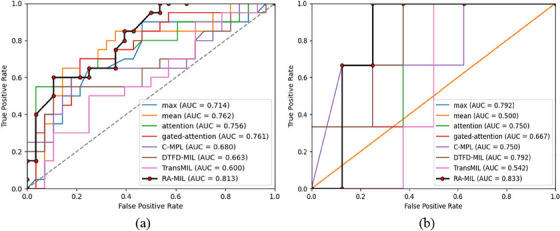
ROC curves of test datasets. (a) Internal test set; (b) public test set. AUC, area under the receiver operating characteristic curve.

To verify the advantage of our proposed MIL‐pooling strategy, our RA‐MIL model is compared with seven SOTA MIL methods, including mean pooling MIL, max pooling MIL, attention MIL,^22^ gated‐attention MIL,^22^ C‐MPL,^14^ DTFD‐MIL,^23^ and TransMIL.^32^ Gated‐attention MIL adds a gating mechanism with the *tanh* non‐linearity to the attention MIL. C‐MPL aggregates instance‐level features into a bag‐level representation by assessing contextual information between adjacent instances. Contextual scope (i.e., the range of contextual slices) in the C‐MPL is empirically set as 3 in our evaluation. DTFD‐MIL randomly builds pseudo‐bags and performs 2 tiers MIL. TransMIL is a Transformer based MIL that explores both morphological and spatial information of instances. We train these models on the training dataset and then separately evaluate them on internal and public test datasets. Comparative results are listed in Table 2, and corresponding ROC curves for two test datasets are displayed in Figure 4. Compared with these MIL methods, the RA‐MIL model achieves the highest *AUC* value on the internal test dataset (*AUC *= 0.809) and public dataset (*AUC *= 0.833). As per the Mann–Whitney *U* test, our RA‐MIL model demonstrates significant improvements (e.g., *p*‐value < 0.05) over the compared methods in most situations. Max‐pooling MIL provides *AUC* values of 0.716 and 0.792. Mean‐pooling MIL provides *AUC* values of 0.762 and 0.500. Attention MIL and gated‐attention MIL also provide acceptable performances, with *AUC* values ranging between 0.667 and 0.761. These results demonstrate the superiority of our RA‐MIL model. Unlike the mean pooling, max pooling, and C‐MPL with pre‐defined and non‐trainable characteristics, the RA‐MIL using the attention mechanism is more flexible and adaptive. Compared with the attention MIL and gated‐attention MIL, the RA‐MIL refines bag‐level feature representations based on our designed ranking attention strategy. The DTFD‐MIL performs poorly on LNM prediction, possibly due to inaccurate labels in some pseudo‐bags, making model training more challenging. The inferior performance of TransMIL, as compared to our method, can primarily be attributed to overfitting issues associated with training transformers on small datasets.

**TABLE 2:** Comparison with other MIL models on LNM prediction. Note that *p*‐values are provided to highlight the difference between the proposed RA‐MIL and other methods in terms of automatic predictions.

Methods	Datasets	*Acc* (%)	*AUC* (95% CI) (%)	*Pre* (%)	*Rec* (%)	*Spe* (%)	F1 (%)	*P*‐value
Max‐pooling MIL	Internal	68.8	71.6 (58.8, 83.8)	58.1	90.0	53.6	70.6	<0.0001
	Public	72.7	79.2 (53.6, 100.0)	50.0	100	62.5	66.7	0.0255
Mean‐pooling MIL	Internal	77.1	76.2 (62.7, 87.3)	76.5	65.0	85.7	70.3	<0.0001
	Public	72.7	50.0 (50.0, 50.0)	0.0	0.0	100	0.0	<0.0001
Attention MIL^22^	Internal	79.2	75.7 (63.0, 87.4)	91.7	55.0	96.4	68.7	<0.0001
	Public	72.7	75.0 (44.4, 100.0)	50.0	100	62.5	66.7	<0.0001
Gated‐attention MIL^22^	Internal	75.0	76.1 (64.3, 87.5)	70.0	70.0	78.6	70.0	<0.0001
	Public	63.6	66.7 (33.3, 100.0)	42.9	100	50.0	60.0	<0.0001
C‐MPL^14^	Internal	72.9	68.0 (54.5, 80.7)	70.6	60.0	82.1	64.9	<0.0001
	Public	90.0	79.2 (40.0, 100.0)	100	66.7	100	80.0	0.0013
DTFD‐MIL^23^	Internal	75.0	66.4 (51.9, 79.5)	78.6	55.0	89.3	64.7	<0.0001
	Public	72.7	79.2 (50.0, 100.0)	50.0	100	62.5	66.7	0.7426
TransMIL^32^	Internal	64.6	60.0 (43.5, 76.5)	58.8	50.0	75.0	54.1	0.0052
	Public	63.6	54.2 (13.9, 94.5)	42.9	100	50.0	60.0	<0.0001
Proposed RA‐MIL	Internal	77.1	80.9(71.6, 90.5)	80.0	60.0	89.3	68.6	–
	Public	81.8	83.3 (57.1, 100.0)	60.0	100	75.0	75.0	–

Abbreviations: *Acc*, accuracy; *AUC*, area under the receiver operating characteristic curve; *Pre*, precision; *Rec*, recall; *Spe*, specificity; F1, F1‐score.

To verify the advantages of our MIL‐based feature learning and aggregation strategies, our RA‐MIL model is also compared with three recent studies on automatic cervical cancer diagnosis. Kan Y et al.^7^ computed Radiomics features from manually annotated tumorous regions in MRI images by using the PyRadiomics package.^33^ Then the top 10 features ranked by the minimum redundancy‐maximum relevance (MRMR) method were used to train a support vector machine (SVM) model to predict LNM status. Hua W et al.^10^ aggregated convolutional features from a VGG‐19 network with radiomics features to train an SVM model, subsequently predicting the LVSI status. Chen J et al.^34^ transformed the CT scans into videos and utilized the pre‐trained Inflated 3D ConvNet video classification network as the architecture. We re‐implement these three methods and train the models with carefully selected parameters by using a training dataset. Table 3 provides comparative LNM prediction results on an internal test set. Note that we only report quantitative comparisons on the internal test set since it manually annotates tumor regions provided by our collaborated radiologists. As observed in Table 3, although the proposed RA‐MIL model does not use any tumor annotations, it still obtains the best result, which has about 7% to 10% improvements in *AUC* values. These results indicate that the proposed RA‐MIL model can more effectively learn radiological features that are informative to LNM diagnosis for cervical cancer patients.

**TABLE 3:** Comparison with automatic cervical cancer diagnosis studies.

Methods	*Acc* (%)	*AUC* (%)	*Pre* (%)	*Rec* (%)	*Spe* (%)	F1(%)
Kan Y. et al.^7^	74.2	70.7	67.4	69.0	76.9	66.6
Hua W. et al.^10^	72.0	70.3	68.4	60.3	78.1	60.4
Chen J. et al.^34^	71.4	73.1	57.7	83.3	64.5	68.2
Proposed RA‐MIL	**77.1**	**80.9**	**80.0**	**60.0**	**89.3**	**68.6**

Abbreviations: *Acc*, accuracy; *AUC*, area under the receiver operating characteristic curve; *Pre*, precision; *Rec*, recall; *Spe*, specificity; F1, F1‐score.

### 3.3 Visualization

**FIGURE 5:**
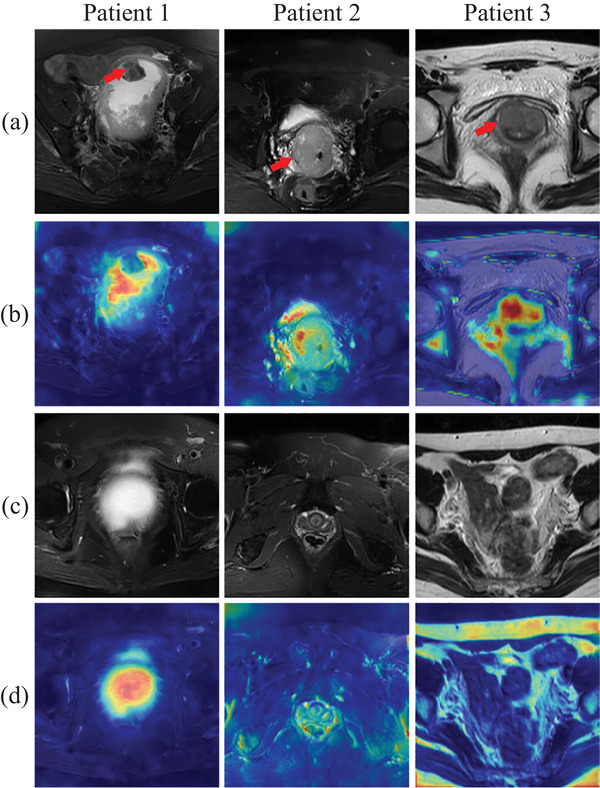
Visualization by using the Grad‐CAM algorithm.^35^ (a) MRI slices with the highest attention scores from three patients; (b) visualization of (a); (c) MRI slices with the lowest attention scores from the same patients in (a); (d): visualization of (c). Red color corresponds to higher gradient values, while blue color corresponds to lower gradient values. Red arrows in (a) point to tumor regions.

The visual interpretability can usually help physicians to understand inference decisions made by the deep learning model. In our RA‐MIL model, MRI slices are separately encoded into feature vectors, and then attention scores are computed for the feature vectors. These attention scores can be considered as contributions of different MRI slices to the LNM prediction. If an MRI slice has a high attention score, it is more informative to predict LNM status, and vice versa. As shown in Figure 5, to interpret model decisions, we apply the Grad‐CAM algorithm^35^ which uses gradients flowing into the final convolutional layer to produce a coarse localization map highlighting important regions in the image for LNM prediction. Figure 5a shows informative MRI images (i.e., with the highest attention scores) of three randomly selected patients, while Figure 5b shows corresponding visualized heatmaps highlighting regions most influential to LNM prediction. To compare informative and uninformative MRI slices, in Figure 5c we display three MRI slices with the lowest attention scores from those three patients, and their heatmaps are shown in Figure 5d. As observed in Figure 5, the heatmaps reveal that our model focuses on tumor and peri‐tumor regions in informative MRI slices (see Figures 5a,b). However, the highlighted regions are more dispersed across uninformative MRI slices, corresponding to the bladder (left), some non‐tumor regions (middle), and the body edge (right) in Figure 5d. Our collaborating radiologists confirmed that the highlighted regions in uninformative slices do not align with what they consider important for LNM diagnosis, or those areas are too broad. This verifies that our model is consistent with the diagnostic decisions made by radiologists. In other words, it focuses more on diagnostic slices and discriminative regions, such as tumors and peri‐tumors, where tumorous morphologies and textures are typically considered relevant for LNM diagnosis.^12^ Visualization results confirm that our RA‐MIL model can identify more informative slices by using the attention mechanism. Furthermore, it confirms that the tumor and its surrounding regions contain the most relevant areas for prediction, consistent with previous research^12^ showing that peri‐tumor regions are also informative for LNM diagnosis.

## 4 DISCUSSION

This study presents a novel RA‐MIL model for predicting the LNM status of cervical cancer patients by using T2 MRI. Four evaluations for model selection are designed to assess different components of the RA‐MIL model. Using all 2D MRI slices as our model input, the ResNet50 as feature extractor along with the proposed ranking‐attention pooling block reaches the best performance in the LNM prediction task. By evaluating training and test MRI cohorts, the RA‐MIL exhibits superior predictive efficacy than other MIL pooling methods and three automatic cervical cancer diagnosis studies. Furthermore, the RA‐MIL model provides well interpretability for LNM prediction due to its attention mechanism.

Feature aggregation to create patient‐level representation is an important component in our RA‐MIL model. Using the MIL ideology, we build the connection between patients (bag‐level) and their MRI slices (instance‐level). Our proposed RA‐MIL has been compared with seven other MIL methods, including traditional MIL and newly developed MIL models. According to the results in Table 2, attention‐based MIL models tend to perform better than non‐attention‐based models (max pooling and C‐MPL^14^) because of self‐adaptive and trainable properties in the attention mechanism. Compared with traditional attention‐based MIL (attention MIL and gate‐attention MIL^22^), our RA‐MIL model improves attention MIL by refining features according to the designed ranking attention strategy, where feature aggregation is self‐adaptive and trainable. In addition, using the RA‐MIL model with the Grad‐CAM algorithm can identify informative MRI slices and important regions to LNM prediction, thus providing the interpretability for our model decision.

Unlike existing relevant studies, the RA‐MIL model can predict LNM status without relying on manual tumor annotations or pre‐defined feature selection. Existing studies adopted tumor regions or peri‐tumor regions for feature extraction or feeding into the deep learning model, and hence tumor ROIs have to be manually contoured by radiologists. We evaluate different MRI input regions and show that the RA‐MIL reaches the best performance when using all T2 MRI slices as the inputs for our model, as illustrated in Figure 3c. In addition, the RA‐MIL model greatly improves over two studies^7^, ^10^ that make predictions using radiomics features of tumor regions as shown in Table 3. This indicates that the attention mechanism used in our model can effectively learn radiological image features from unannotated MRI slices, which can reduce manual annotation burdens typically required in traditional machine learning models. Our results support that informative regions to LNM prediction not only appear in tumor regions, peri‐tumor regions, or tumor slices, which might indicate that comprehensive information contained in 3D MRI is important in LNM prediction.

Our research highlights the significant potential of deep learning models in the diagnosis of cervical cancer LNM from T2 MRI. Compared with other contrast‐enhanced sequences, the T2 sequence is more practical in medical institutions with limited resources. The promising results from experiments on the T2 sequence support the potential of our model for broader applications in general clinical scenarios. Multicenter datasets from one clinical center and one open‐source set are obtained to independently verify the generalization capabilities of the proposed model. Although data harmonization might be necessary if there indeed exist severe color variations among MRI images from different centers,^36^ our experiments show acceptable performance on two distinct test datasets and reveal the feasibility of our proposed method for application in multicenter scenarios. Interpretability is becoming increasingly crucial for deep learning‐powered applications, especially in healthcare and medical studies.^37^ Our research offers visualizations on each T2 MRI slice for interpretability in LNM diagnosis, as illustrated in Figure 5. The RA‐MIL could automatically rank MRI slices according to attention scores and generate heatmaps to identify informative regions, thereby enhancing the interpretability of the proposed method and making model predictions more transparent.

**FIGURE 6:**
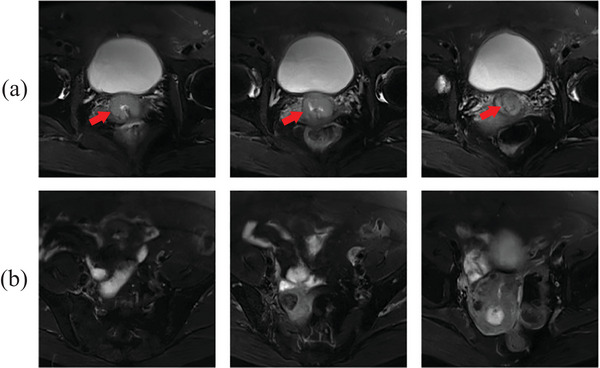
A failure example by our RA‐MIL. (a) MRI slices containing the tumor but excluded by our RA‐MIL. (b) MRI slices included by our RA‐MIL, but without tumor tissue. Red arrows in (a) point to tumor regions.

Although our RA‐MIL model provides a promising LNM prediction performance, there are still some limitations in this retrospective study. T2 MRI is a basic scanning in MRI protocol, so research based on T2 is much easier to promote to different clinical centers. However, we notice some other MRI sequences, such as contrast‐enhanced T1‐weighted imaging (CET1),^7^, ^12^ diffusion‐weighted imaging (DWI),^38^ and apparent diffusion coefficient (ADC) maps,^12^ which might be options to predict LNM status. Thus, it might be beneficial to apply multimodal MRI data to enhance the prediction performance of our RA‐MIL model. In addition, we attempt to test model generalizability using a public TCGA dataset, but it is difficult to verify the robustness of our model on such a small dataset. Therefore, data from more clinical centers are beneficial for verifying the reproducibility of our proposed model. There are also failure cases due to the exclusion of important slices in our method. Figure 6 presents an example of an LNM‐positive case, that is, incorrectly predicted by our RA‐MIL but correctly identified by the attention MIL using all slices. This case features a relatively small tumor, with only 3 out of 20 slices containing tumor tissue (see Figure 6a). The RA‐MIL fails to identify the patient as LNM‐positive, as all the selected slices lack tumor tissue (see examples in Figure 6b). This suggests that our RA‐MIL approach may encounter difficulties in accurately diagnosing cases with very small tumors.

## 5 CONCLUSIONS

In this study, we develop an RA‐MIL model for LNM prediction using multicenter T2 MRI of cervical cancer patients. Our end‐to‐end network achieves remarkable classification performances even without any manual ROI annotations or radiomics feature selection. Built on the MIL ideology and attention mechanism, the RA‐MIL model can directly aid in patient‐level LNM diagnoses and recognize informative MRI slices in LNM‐positive patients. Both informative MRI slices and crucial regions can be detected to explain the model decisions of LNM diagnosis. The proposed model serves as a non‐invasive tool for the preoperative LNM diagnosis in cervical cancer patients. Our work further demonstrates the superiority of the deep learning method employed in MRI image analysis and highlights its potential for transferability to other diagnostic tasks.

## AUTHOR CONTRIBUTIONS

All authors contributed to the study's conception and design. Research design: Shan Jin, Hongming Xu, and Yue Dong. Data collection and labeling: Yue Dong and Fengying Qin. Model design and data analysis: Shan Jin, Xinyu Hao, and Ranran Wang. The first draft of the manuscript: Shan Jin. Revision of manuscript: Hongming Xu, Xiaofeng Wang, and Fengyu Cong. All authors commented on previous versions of the manuscript. All authors read and approved the final manuscript.

## CONFLICT OF INTEREST STATEMENT

The authors declare no conflicts of interest.

## Supporting information



SUPPORTING INFORMATION
