# Supplementary material for: Ranking attention multiple instance learning for lymph node metastasis prediction on multicenter cervical cancer MRI
Source: J Appl Clin Med Phys. 2024 Oct 6;25(12):e14547. doi: 10.1002/acm2.14547 (PMC11633800; doi:10.1002/acm2.14547)
Supplement: Supplementary file 1 — SUPPORTING INFORMATION [file ACM2-25-e14547-s001.docx]

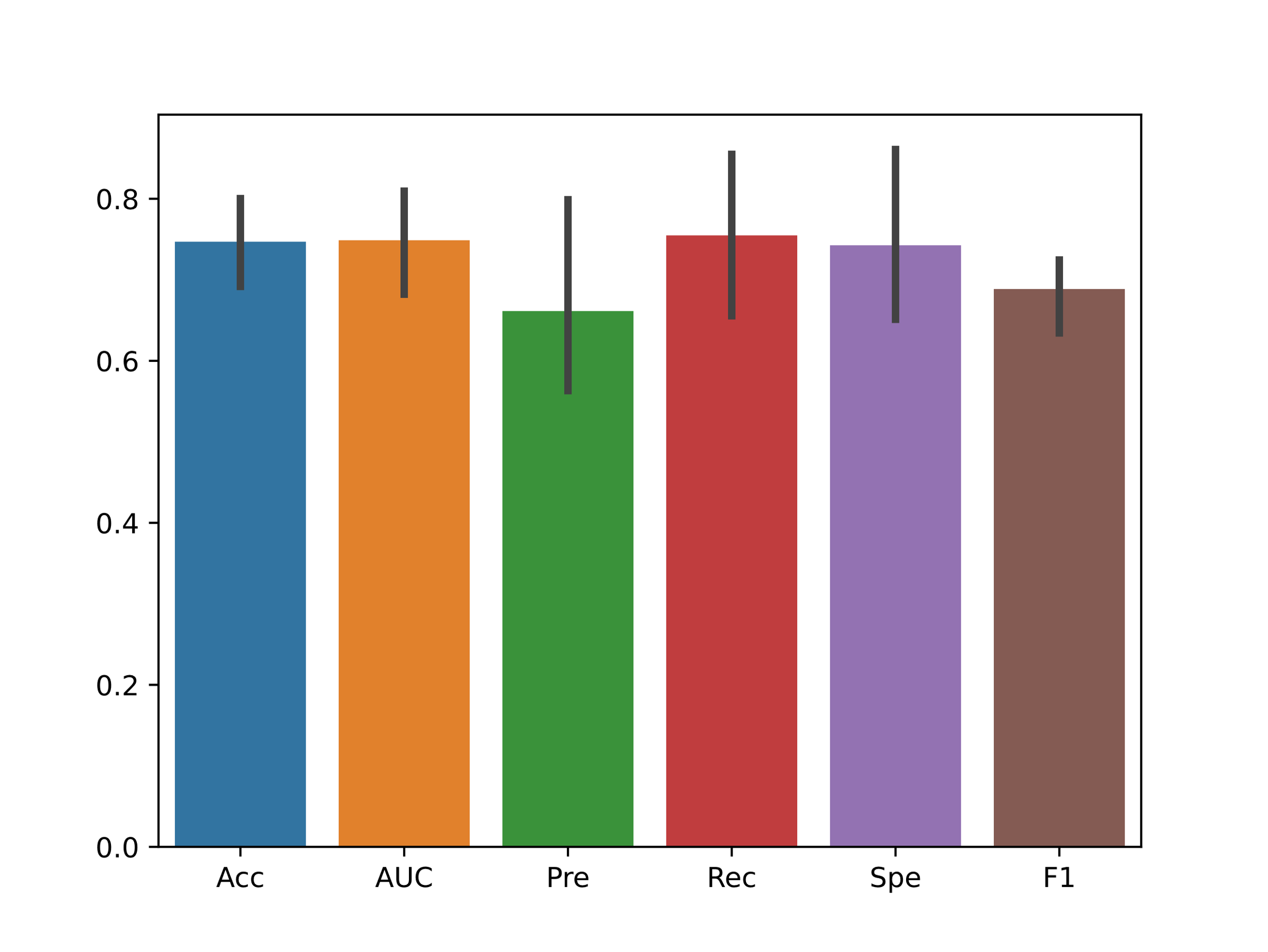


**Fig. S1** The results of 5-fold cross-validation on the training dataset, where the average values and standard deviations are computed. Specifically, the cross validation achieves the AUC of 0.749 ± 0.074, Acc of 0.747 ± 0.058, Pre of 0.661 ± 0.135, Rec of 0.755 ± 0.119, Spe of 0.743 ± 0.122, and F1 score of 0.689 ± 0.054. Overall, our internal cross-validation results verify the feasibility of using our RA-MIL model for LNM prediction from T2 MRI, even without using any annotations on tumor regions or other ROIs

Acc: accuracy, AUC: area under the receiver operating characteristic curve, Pre: precision, Rec: recall, Spe: specificity, F1: F1-score
